# Knowing What’s Out There: Awareness of Non-Alcoholic Fatty Liver Disease

**DOI:** 10.3389/fmed.2014.00004

**Published:** 2014-03-24

**Authors:** Vishal Ghevariya, Nan Sandar, Kishor Patel, Nehal Ghevariya, Ruchit Shah, Joshua Aron, Sury Anand

**Affiliations:** ^1^Division of Gastroenterology, Elmhurst Hospital Center, Icahn School of Medicine at Mount Sinai, Elmhurst, NY, USA; ^2^Division of Gastroenterology, The Brooklyn Hospital Center, Weill Cornell Medical School, Brooklyn, NY, USA

**Keywords:** fatty liver, non-alcoholic fatty liver disease, cirrhosis, liver cancer, steatosis, steatohepatitis

## Abstract

**Background:** Non-alcoholic fatty liver disease (NAFLD) is the most common hepatic disorder, which poses a significant health burden in the western countries. As the epidemic of obesity slides health downward, the incidence of NAFLD is evidently increasing.

**Aim:** We aimed to ascertain the awareness of NAFLD and its risk factors in the general population, which may be helpful in designing educational tools to promote prevention, early detection, and treatment of this disorder.

**Methods:** A survey of 5000 non-institutionalized residents of Brooklyn, NY, USA was conducted. Sixteen items were included in the survey questionnaire including awareness of fatty liver, predisposing factors of NAFLD, awareness of cirrhosis, and conditions that advance to cirrhosis. The questionnaire also addressed awareness of prevention, diagnostic methods and treatment of NAFLD, and education of physicians to their patients about NAFLD.

**Results:** Overwhelming majority of the subjects was not aware of NAFLD and stated that their physicians did not have a discussion about NAFLD.

**Conclusion:** Non-alcoholic fatty liver disease is a preventable liver disorder with limited treatment options. Thorough counseling by primary care physicians can be of paramount importance in preventive strategy for NAFLD. We should target our teenage population in an era of obesity epidemics of all times.

## Introduction

Non-alcoholic fatty liver disease (NAFLD) consists of a spectrum of liver pathology ranging from steatosis to steatohepatitis, fibrosis, cirrhosis, and ultimately hepatocellular carcinoma. In United States, chronic liver disease and cirrhosis are the tenth leading cause of death. Metabolic syndrome with its manifestation as non-alcoholic fatty degeneration is a silent assassin of many organs including the liver (1–5). As the epidemic of obesity slides health downward, the incidence of NAFLD is evidently increasing (1). Two factors responsible for increased prevalence of this condition are: increasing obesity and the practice of measuring liver function tests before starting statin therapy (1). NAFLD is the most common cause of elevated transaminases and cirrhosis of liver. This disorder is often only suspected and investigated in those with abnormal liver function tests. However, a recent study demonstrated that patients could have histological changes of NAFLD with or without abnormal liver tests. Interestingly, prevalence of advanced fibrosis was similar between the groups with and without abnormal liver tests (2).

Non-alcoholic fatty liver disease affects 10–24% of the general population in the world. Reported prevalence increases up to 75% in obese individuals. NAFLD affects 2.6% of children and up to 50% of obese children (3, 4). A combination of diabetes and obesity may pose an added risk. Up to 100% of obese diabetics have at least mild steatosis, 50% have steatohepatitis, and 19% have cirrhosis (6, 7).

The prevalence of NAFLD in United States was recently estimated using ultrasonographic assessment of hepatic steatosis (8). This study revealed hepatic steatosis in 21.4% and NAFLD in 19% of the studied population (8). Obesity affects 22.5% of people 20 years or older (9). Steatosis is found in over two-thirds of the obese population, regardless of diabetic status. More than 90% of morbidly obese persons have steatosis. Steatohepatitis affects 3% of lean population, 19% of obese, and up to 50% of morbidly obese persons (10). Thus, up to 40 million obese individuals may have changes consistent with NAFLD. Diabetes mellitus affects 7.8% of US population over the age of 20 years (11). Up to half of these individuals have NAFLD (8, 12).

Mild to moderate elevation of serum transaminases is often the only laboratory abnormality found in NAFLD. When this condition advances to cirrhosis, other abnormalities, including increased alkaline phosphatase and γ-glutamyltransferase, prolonged prothrombin time, hypoalbuminemia, and hyperbilirubinemia may be found. Ultrasonography of the liver may demonstrate increased echogenicity of liver parenchyma and cirrhosis.

Non-alcoholic fatty liver disease is increasingly recognized as a major cause of liver damage (13). Body weight is directly proportional to the likelihood of acquiring NAFLD. With increasing prevalence of obesity in United States, it is an important public health issue. No medications have been proved to directly reduce or reverse liver damage. Gradual weight loss with appropriate glycemic and lipid control is helpful (14). Several studies demonstrated NAFLD as independent risk factors for systemic cardiovascular disease. Increased risk of coronary artery disease, prominently in women is associated with NAFLD (15). A recent study showed NAFLD in 45–54 years age group is a strong independent risk factor for cardiovascular death (16). Another study demonstrated 13% increase in carotid intima–media thickness and increased plaque formation in patients with NAFLD (17).

The pathophysiology, diagnosis, and treatment of NAFLD have been vastly investigated but public awareness of NAFLD is unknown. The aim of this study is to ascertain awareness of NAFLD and its risk factors in the general population, which may be helpful in designing educational tools to promote prevention, early detection, and treatment of this disorder.

## Materials and Methods

**Figure 1:**
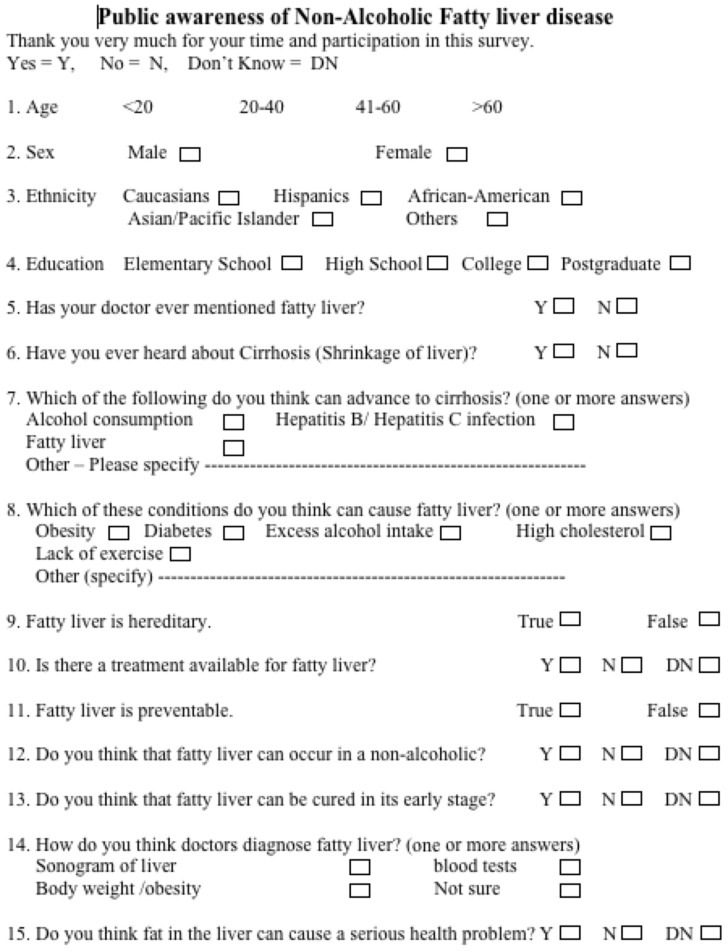
**Survey questionnaire**. NAFLD, non-alcoholic fatty liver disease.

From March 2008 to July 2008, members of the research study group conducted a written survey with the residents of Brooklyn, NY, USA. This cross-sectional survey was conducted in public places such as shopping centers, deli stores, and coffee shops. All participants were 18 years or older. To achieve the target of 5000 respondents, 6849 individuals were approached to participate in a semi-structured interview followed by answering the survey questionnaire and 1849 declined to participate. After the subject agreed to participate and fill out the survey, our surveyor indicated that fatty liver is fat deposition in the liver and cirrhosis is when liver failure begins. After this briefing, the subject was allowed to complete the questionnaire (Figure 1). The study group of GI faculties, hepatologists, fellows, medical residents, statisticians, and nutritionists developed a comprehensive questionnaire. The questionnaire was refined after discussion with a focus group. The average time burden for filling out the questionnaire was approximately 2 min.

Sixteen items were included in the survey questionnaire: demographics, level of education, awareness of fatty liver, awareness of cirrhosis, conditions that can advance to cirrhosis, predisposing factors of NAFLD. The questionnaire also addressed awareness of prevention, diagnostic methods and treatment of NAFLD, and education by physicians to their patients about NAFLD.

## Measurements

Data collected were entered into Analyse-it v2.05 software (Analyse-it Software Ltd, Leeds, UK). Data entry errors were resolved by reference to data collection sheets. Pearson’s chi-square was conducted using Analyse-it v2.05 to explore statistical significance regarding public awareness of fatty liver among different age groups, gender, or education level.

The institutional review board of The Brooklyn Hospital Center, Brooklyn, NY, USA approved the study protocol. Selected survey responses are shown in Table 1.

**Table 1:** **Selected survey responses**.

Public awareness of non-alcoholic fatty liver disease	No	%
Age (years)
<20	604	12
21–40	1989	40
41–60	1634	33
>60	773	15
Education
Elementary school	2022	41
High school	1790	36
College	869	17
Postgraduate	319	6
Has your doctor ever mentioned fatty liver?		
No	4923	98
Yes	77	2
Have you ever heard about cirrhosis?		
Yes	989	20
No	4011	80
Which of the following you think can advance to cirrhosis?		
Alcohol abuse	309	6
Hepatitis	327	6.5
Fatty liver	126	2.5
Other	3	0.06
Do not know	4235	84.7
Which of these conditions you think can cause fatty liver		
Obesity	394	8
Diabetes	242	5
Alcohol abuse	43	0.7
High cholesterol	90	1
Lack of exercise	57	1
Other	21	0.3
Do not know	4153	83
Is fatty liver hereditary?		
Yes	3525	70
No	1475	30
Is there a treatment available for fatty liver?		
Yes	64	1
No	40	1
Do not know	4896	98
Fatty liver is preventable		
True	96	2
False	259	5
Do not know	4645	93
Do you think fatty liver can be reversible in its early stage?		
Yes	105	2
No	90	2
Do not know	4805	96
Do you think fat in the liver can cause a serious health problem?		
Yes	211	4
No	53	1
Do not know	4736	95

## Results

Study population comprised of 52% males and 48% females. Forty percent were 20–40 years old, 33% were 41–60 years old, 15% were more than 60 years old, and 12% were <20 years of age. Fifty three percent self-reported as African-American, 33% Caucasians, 10% Hispanics, and the remaining 4% were Asians and other ethnic groups.

**Figure 2:**
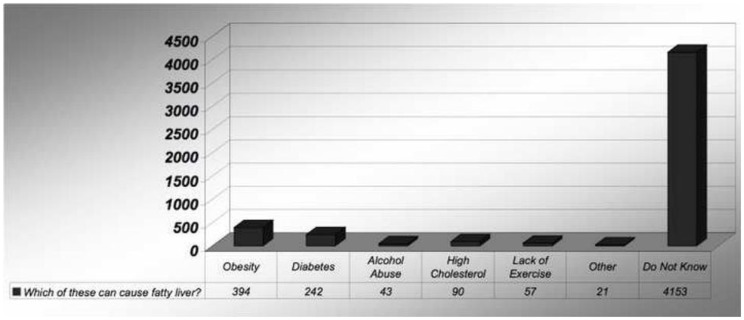
**Awareness of etiologies of NAFLD**.

In fact, 98% of the study population stated that their physicians did not have a discussion regarding NAFLD. Only 20% of the respondents have heard about cirrhosis of liver. Upon further questioning, 84% of the subjects stated that they were not aware of the conditions that can potentially cause NAFLD or cirrhosis of the liver respectively (Figure 2).

Overwhelming majority (70%) of the study population believed that NAFLD is hereditary and only 2% the population recognized NAFLD as a preventable condition. Similarly, 96% of the subjects were unaware of the reversibility of NAFLD in its early stage. Only 5% of the subjects thought that NAFLD could occur in a non-alcoholic individual.

**Figure 3:**
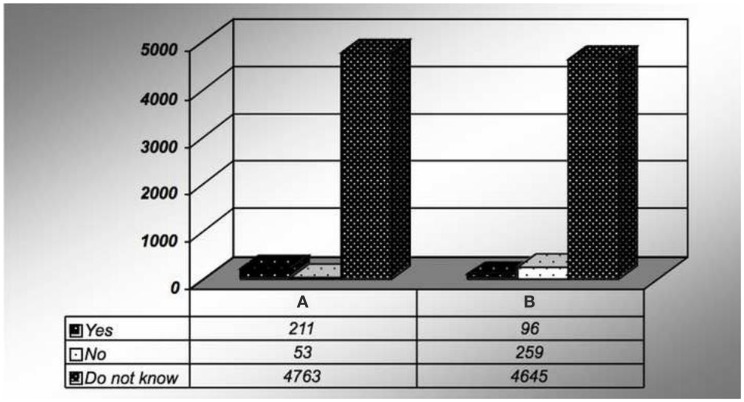
**Survey response for (A) fatty liver is preventable**. And **(B)** do you think fat in the liver can cause a serious health problem?

Ninety-three percent of the subjects were not sure how this condition is diagnosed. Ninety-five percent did not feel that fat deposition in the liver could cause serious health problems (Figure 3). Similarly, 93% did not acknowledge the components of metabolic syndrome.

## Subgroup Analysis

There was no statistically significant difference between male and female regarding awareness of NAFLD. Similarly, both men and women thought that fatty liver is hereditary. Participants with lower education level (up to the high school) were aware of cirrhosis more than the subjects with advanced degrees (*p* < 0.0001) and knew that alcohol drinking could lead to cirrhosis (*p* < 0.0001). There was no significant difference between age groups of <20 years old and more than 20 years old regarding knowledge of cirrhosis (8 vs. 889, *p* = 0.8625). This finding remained true for subgroup analysis of ages <20, 21–40, 41–60, and above 60 years (20.64% “yes” responses vs. 79.36% “no” responses, *p* = 0.012). There is no statistical difference between responses by males and females regarding their knowledge of cirrhosis [502, 10.04% (males) vs. 487, 9.78% (females) for “yes” response].

Similar percentage of subjects with higher education thought that alcohol and hepatitis could lead to cirrhosis. Study population of <20 year of age reported significantly less interaction with their physicians in discussing about fatty liver (8 vs. 889, *p* < 0.001). After excluding “not sure” responses, respondents of <20 years of age reported sonogram as the method for diagnosing fatty liver at a greater frequency (*p* < 0.05).

## Discussion

Non-alcoholic fatty liver disease is not a benign disease. Progressive liver biopsies have shown histological progression of steatosis into fibrosis in 32%, development of cirrhosis in 20%, and liver-related death in 12% of patients over 10 years (18). Pathologic effects of this disorder also extend to the cardiovascular system (15, 16). Conditions such as diabetes mellitus, obesity, dyslipidemia, hypertension, and oxidative stress play a critical role in pathophysiology of this condition. NAFLD, with its potential to affect younger, non-obese, and non-diabetic individual is a major public health problem. Widely approached therapy of this disorder remains weight loss and appropriate glycemic and lipid control (19). No treatment has proved to ameliorate or to prevent progression of NAFLD (20). Higher overall and liver-related mortality is observed in general US population with NAFLD (18). Recent studies proposed use of vitamin E and pioglitazone in the treatment of NAFLD (21). Therefore, preventive measures are the cornerstone to tackle this soaring public health problem.

This study assessed the knowledge and attitude of the general population toward NAFLD and its precipitating factors. Overwhelming majority of the participants had little awareness of NAFLD regardless of their age, gender, or educational status. Vast majority of them stated that their physicians never mentioned NAFLD to them but respondents of <20 years of age reported significant less interaction with their physicians. A recent study demonstrated insufficient knowledge of primary care physicians about NAFLD (22). Respondents of all age groups reported obesity as the culprit for NAFLD but they were not aware of the other risk factors for NAFLD. Different levels of education had different opinions about risk factors for cirrhosis. Respondents of lower education reported that they were aware of cirrhosis and alcohol could lead to cirrhosis. The majority of respondents reported that fatty liver was hereditary. This implies the public misperception of this disease and its importance in implementing tools for public education.

The limitations of the study include the possibility of sampling errors. As with most cross-sectional surveys, our study population may not be entirely representative of the general population. Respondents had a chance to choose more than one answers in some questionnaires in the survey but majority of the participants chose only one answer.

## Conclusion

Our study proved the fact that surprisingly higher percentage of our general population remains unrecognized of this silent but concerning disease and patient education programs were lacking. We hope that this study will draw attention for the urgent need for an education campaign among physicians and the general population.

Awareness of NAFLD must be promoted for prevention, early detection, and treatment. Thorough counseling by primary care physicians can be of paramount importance in preventive strategy for NAFLD. Educational tools including mass media should be utilized to increase awareness of NAFLD.

## Conflict of Interest Statement

The authors declare that the research was conducted in the absence of any commercial or financial relationships that could be construed as a potential conflict of interest.
